# Service transitions, interventions and care pathways following remittal to prison from medium secure psychiatric services in England and Wales: national cohort study

**DOI:** 10.1192/bjo.2020.62

**Published:** 2020-08-03

**Authors:** Sarah-Jayne Leonard, Roger T. Webb, Jennifer J. Shaw

**Affiliations:** Offender Health Research Network, Centre for Mental Health and Safety, University of Manchester, UK; Centre for Mental Health and Safety, University of Manchester, UK; Centre for Mental Health and Safety, University of Manchester, UK

**Keywords:** Forensic mental health services, human rights, prison mental health, Mental Health Act, offender pathway

## Abstract

**Background:**

Little is known internationally about return to prison from in-patient psychiatric services, including: circumstances leading to return, aftercare services and subsequent patient outcomes.

**Aims:**

To examine and describe: (a) circumstances leading to return to prison from medium secure services; (b) available aftercare and early outcomes of returned persons; and (c) implications for policy development.

**Method:**

Prospective cohort design with all patients (*n* = 96) returned to prisons from 33 National Health Service (NHS) medium secure services over a 6-month period in England and Wales. Follow-up was conducted for 1 year post-remittal, across 60 prisons.

**Results:**

Less than 20% of patients with legal entitlement to section 117 aftercare under the Mental Health Act 1983 were receiving care managed/delivered via the care programme approach. Subsequent pathways included: inter-prison transfer (30%), use of the Assessment, Care in Custody and Teamwork process (49%), referral to secure services (21%) and community release (30%). Less than half of community releases were referred to a community mental health team.

**Conclusions:**

Findings suggest that persons returned to prison are a vulnerable group of patients, many of whom require intervention (e.g. enhanced monitoring, admission to a healthcare wing, readmission to secure mental health services) on return to prison in the absence of targeted aftercare services. More robust guidance for discharge and aftercare planning procedures for persons remitted to prison should be developed to ensure that the benefits of in-patient admission are maintained and that individuals’ legal rights to ongoing aftercare are upheld.

The international prison population is estimated to be in excess of 11 million.^1^ Prevalence of psychiatric disorders within this population is high.^2,3^ For those in need of acute in-patient care, transfer to forensic mental health hospitals is required to provide treatment and therapeutic intervention.^4–6^ In many high-income countries, hospital transfer procedures are supported by mental health legislation that protects an individual's rights to quality treatment and care.^7^ There is a current international drive to modernise forensic mental health services^8–10^ focusing on patients’ rights through the development and implementation of robust healthcare standards.^11^ There exists a large body of research investigating patient outcomes following transition from secure services, which highlights the importance of robust ongoing treatment post-discharge and bespoke community aftercare arrangements.^12^ In contrast, there is little research internationally on the process of discharge, aftercare and outcomes for those returned to prison following treatment. This is a key transition point in an individual's care pathway. This paper focuses on the remittal care pathway in England and Wales (those returned to prison from in-patient psychiatric care) and addresses clinical issues that are experienced across many countries (details available from S.-J.L. on request).

## Secure care in England and Wales

At any given time it is estimated that forensic secure psychiatric services work with approximately 8000 people, at a cost of £1.2 billion for the National Health Service (NHS) in England and Wales.^13^ These services operate at three levels (low, medium and high), providing a range of physical, procedural and relational security measures to ensure effective treatment. The focus of this research is on medium secure services. Progress and transition through medium secure services should be determined by reduction in risk of harm to others and reduced need for care and supervision.^14^ Final discharge decisions are formally agreed for detained patients under the auspices of the care programme approach (CPA),^15,16^ which requires that a meeting (section 117 of the Mental Health Act 1983 (MHA)) is held prior to discharge. For those discharged into the community, an individual's appointed care coordinator manages transition and discharge, utilising risk assessment and proactive risk management strategies to ensure recovery and rehabilitation.^14,17^ This process should be the same when discharge is to prison, with prison mental health services ‘actively participating’ in the remission process by developing a CPA care plan to outline ongoing mental health services input required.^14^ Guidance for remittal was first outlined in 2005 within the Offender Mental Health Care Pathway.^18^ Since then, there has been no formal evaluation of the remittal guidance. The recent independent review of the MHA proposed the process of discharge and aftercare from in-patient services as a priority area, and specifically highlighted the need for reform of the process of prison remittal.^19^ Since May 2019, NHS England has also been in open consultation regarding the current procedural practice of remittal.^19^ However, to date, no detailed good practice guidance for effective remittal and ongoing care exists.

## Remittal to prison

Remittals to prison from medium secure services increased in frequency during 2010–2011 and were observed to represent over 20% of all discharges.^21,22^ Persons who are remitted to prison are a vulnerable group of psychiatric patients, two-thirds of whom have a primary diagnosis of severe mental illness (SMI). In comparison to patients discharged via a community care pathway, those remitted to prison experience significantly more psychotic symptoms at time of discharge and are assessed both to be at a significantly higher risk of future violence and to have a lower prevalence of protective factors that mitigate subsequent risks of offending and relapse, as measured by the Historical Clinical Risk Management-20 (HCR-20) version 3^23^ and Structured Assessment of Protective Factors for Violence Risk (SAPROF)^24^ respectively. This transition is a time of elevated risk and vulnerability and it is unclear what degree of aftercare is available in the prison estate for those returning from an admission to medium secure services. The current demand for prison mental health services and lack of adequate resources is challenging for prison healthcare providers, and the most recent review of standards for mental health in-reach teams (MHITs) highlighted that there is substantial variability between prisons, lack of integrated working, and shortcomings in ensuring continuity of care between prisons and on release.^25,26^ Although the specification for prison mental health services is continually updated,^13^ there remains concern over the quality of prison mental healthcare provision and a long-awaited need to improve service responsiveness and partner relationships.^27^ At present a national targeted post-discharge service specification for prison remittals in the UK does not exist.

This is the first study internationally to examine the remittal care pathway from secure services, with the aim of obtaining evidence to inform the development of good practice guidance for secure and prison mental health services, regarding safe and effective remittal to prison.

## Method

### Study design

A national prospective-cohort design with a 1-year follow-up was implemented.

### Ethical approval

An application was submitted and accepted by the Confidential Advisory Group, on behalf of the Secretary of State for Health, to conduct the study as a confidential inquiry under section 251 of the National Health Service Act 2006. This allowed for the processing of NHS patient-identifiable information without the individuals’ consent. The authors assert that all procedures contributing to this work comply with the ethical standards of the relevant national and institutional committees on human experimentation and with the Helsinki Declaration of 1975, as revised in 2008. All procedures involving human participants were approved by the North West England Multi Site Research Ethics Committee (09/H1016/126).

### Participants and research sites

Participants were a population of patients remitted to prison from 33 NHS medium secure services in England and Wales over a 6-month period. Eligible patients were those originally admitted/transferred to medium secure services directly from a prison establishment for assessment/treatment, i.e. emergency transfers of both remand (sections 48 and 49 of the MHA) and sentenced prisoners (ss. 47 and 49 MHA). Patients subject to court orders and admitted to medium secure services directly from a prison establishment were also eligible for inclusion (ss. 38 and 45A MHA) (see supplementary Table available at https://doi.org/10.1192/bjo.2020.62 for MHA sections for patients concerned in criminal proceedings or under sentence). There were 96 eligible patients, remitted to 48 prisons. Approvals were gained and medical notes were obtained for patients at 44 prisons. Four prisons were not accessed owing to: governor's refusal (*n* = 1), MHIT's refusal (*n* = 2) and unavailable patient notes (*n* = 1). Inter-prison transfers resulted in an approach to an additional 15 prison sites. Approvals were gained and medical notes were obtained for patients at 12 of these prisons. Three prisons could not be accessed post-transfer owing to the governor's refusal.

### Data extraction

#### Baseline

Demographic, clinical and criminological data were extracted from patients' medical records held by medium secure services. This paper presents patient legal status and discharge circumstances only. Data on full baseline characteristics will be reported in subsequent publications.

#### Follow-up

A structured data collection pro forma allowed for efficient capture of data according to three distinct areas of interest: (a) legal and discharge pathways, (b) MHIT, treatment and access to professionals and (c) management interventions (Appendix).

### Procedure

#### Baseline

The study was conducted concurrently across the 33 medium secure units, with each unit providing notification of planned and actual remittals on a fortnightly basis. Medical records of each patient were accessed via the NHS healthcare provider. The prospective nature of the study enabled cross-checking of missing data or discrepancies with administrators and clinicians.

#### Follow-up

**Fig. 1:**
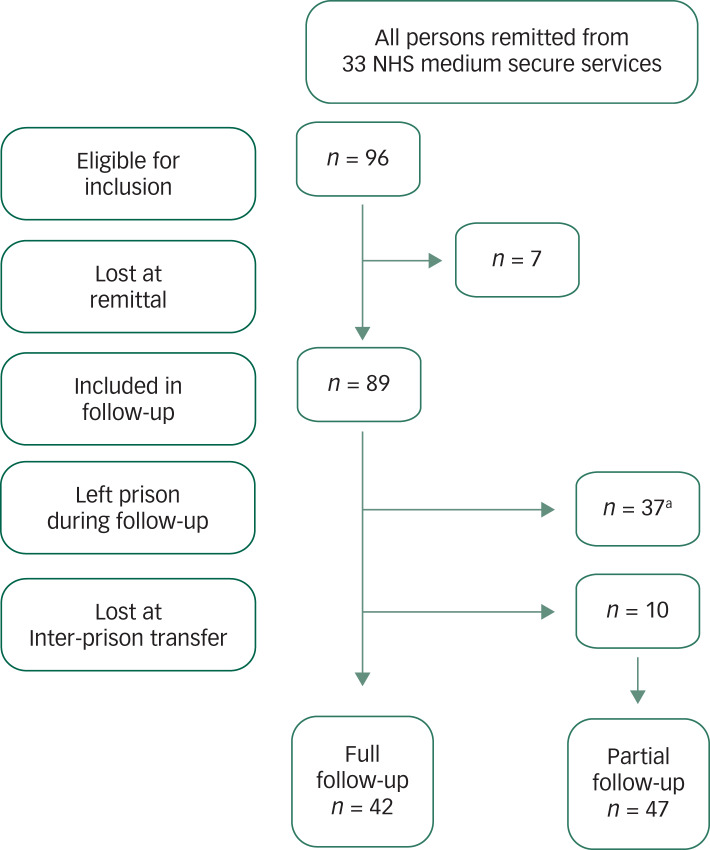
Follow-up of those remitted to prison, outlining reasons for partial follow-up.

Data were extracted from each patient's medical record contained in the SystmOne medical record system (including daily nursing and clinical staff records of patient observations; clinical letters; CPA documentation, etc.). Full follow-up information was ascertained for 42 patients who remained in prison throughout the 1-year period following their remittal to prison. Only partial follow-up information was collected for 47 patients for the following reasons: released from prison (*n* = 27), transferred back to hospital (*n* = 10) and medical-record access problems (as described above, *n* = 10). Thus, at least some follow-up information was available for the great majority (*n* = 89; 93%) of the cohort (Fig. 1).

NHS, National Health Service. a. Readmitted to hospital, *n* = 10; released into the community, *n* = 27.

### Data analysis

Data were analysed using Statistical Package for the Social Sciences (SPSS) for Windows version 22 (IBM Corp, 2013). Frequencies were calculated to describe the characteristics of the sample (legal status and reason for remittal). Chi-squared statistic values were generated to compare the distributions of dichotomous variables across diagnostic groupings (‘primary severe mental illness’, ‘primary personality disorder’ and ‘other’) at follow-up and risk ratios were also reported.

## Results

### Baseline: legal status and circumstances of remittal

We identified and examined 96 eligible persons remitted over the 6-month baseline period; 71 (74%) were subject to a custodial sentence and 25 (26%) were on remand/awaiting sentencing at the time of their remittal.

#### Remand/pre-sentence (*n* = 25)

Over half of persons with remand/pre-sentence status at the time of remittal were directed following court recall (60%, *n* = 15); 11 received custodial sentences at court and 4 were re-remanded to await trial (for 2 of these patients this was in opposition to clinical opinion). The hospital psychiatrist (responsible clinician under s. 34 MHA) directed the remittal for the remaining ten persons (40%) who were remand/pre-sentence status. Six were remitted owing to treatment completion to await trial, two owing to the clinical team not detecting evidence of symptomatology that would warrant detention in medium secure services, and two owing to not engaging with treatment/therapy.

#### Sentenced (*n* = 71)

The responsible clinicians directed the remittal for 70 patients with sentenced status at time of remittal (98%); the remaining person was recalled via the court process. Over half (*n* = 38/71) of sentenced patients were remitted owing to treatment completion to continue their custodial sentence, 7 were remitted owing to the responsible clinician not detecting evidence of symptomatology that would warrant detention in medium secure services, 15 were remitted owing to non-engagement and 10 owing to presenting as too ‘high risk’ to continue to be detained in medium secure services. Sixteen of those with sentenced status at time of remittal were documented as being eligible for parole (*n* = 8) or as being close to their earliest release date (ERD, *n* = 8). Twenty-three (32%, *n* = 23/71) of those with sentenced status at time of remittal were subject to an indeterminate sentence for public protection (IPP).

See the supplementary material for a figure depicting patient legal pathway under the MHA and remittal circumstances.

### Follow-up: access to aftercare and treatment

The follow-up included 89 persons remitted, coded into three distinct diagnostic categories:

- primary SMI (*n* = 35) (including: schizophrenia, paranoid and other psychotic disorders, bipolar disorder and schizoaffective disorder)
- primary personality disorder (*n* = 36) (including all subtypes of personality disorder)
- other (*n* = 18) (common mental health diagnoses, including: depression, obsessive–compulsive disorder, alcohol dependency syndrome, dysthymia and **‘**no current diagnosis/undiagnosed’).

Whole-sample findings are presented in the text and findings by diagnostic category are presented in Tables 1 and 2.

**Table 1:** Aftercare, intervention and inter-prison transfer and release following remittal by diagnostic grouping

	*n* (%)			
	Severe mental illness (*n* = 35)	Personality disorder (*n* = 36)	Other (*n* = 18)	Total *(N* = 89)
*Post-remittal aftercare*				
Right to section 117 aftercare under MHA	32 (91)	30 (83)	16 (89)	78 (88)^a^
Under CPA process during follow-up	8 (23)	7 (19)	2 (11)	17 (19)
Referral to MHIR at remittal prison	34 (97)	35 (97)	18 (100)	87 (98)
Accepted onto MHIR at remittal prison (of those referred, *n* = 87)	29 (85)	24 (69)	12 (67)	65 (75)^b^
Discharged from MHIR at remittal prison (of those accepted, *n* = 65)	1 (3)	3 (13)	4 (33)	8 (12)^c^
*Nature of care received during follow-up*				
Appointment with a psychiatrist	28 (80)	23 (64)	10 (56)	61 (69)
Appointment with a mental health nurse	34 (97)	33 (92)	18 (100)	85 (96)
Appointment with a social worker	3 (9)	2 (6)	3 (17)	8 (9)
Appointment with an occupational therapist	7 (20)	5 (14)	2 (22)	16 (18)
Appointment with a psychologist	1 (3)	3 (8)	–	4 (4)
Prescribed psychiatric medication	33 (94)	28 (78)	13 (72)	74 (83)
Discontinued psychiatric medication in opposition to clinical opinion (of those prescribed, *n* = 74)	16 (48)	15 (54)	4 (31)	35 (47)^d^
Enrolled onto a substance misuse programme	4 (11)	16 (44)	2 (22)	22 (25)
In receipt of therapy	–	2 (6)	–	2 (2)
*Post-remittal intervention*				
Under ACCT process	15 (43)	20 (56)	9 (50)	44 (49)
Once	13 (37)	10 (28)	8 (44)	31 (35)
Twice	2 (6)	7 (19)	1 (5)	10 (11)
Three times	–	2 (6)	–	2 (2)
Four times	–	1 (3)	–	1 (1)
Healthcare wing admission for psychiatric needs	14 (40)	12 (33)	7 (39)	33 (37)
Re-referral to secure psychiatric care, level	9 (26)	8 (22)	2 (11)	19 (21)
HSU referral	1 (3)	3 (8)	–	4 (4)
MSU referral	8 (23)	5 (14)	2 (11)	15 (17)
MSU admission	5 (14)	5 (14)	–	10 (11)
Segregation stay	6 (17)	10 (28)	3 (17)	19 (21)
Self-harm/suicide attempt ^e^	6 (17)	17 (47)	4 (23)	27 (30)
*Inter-prison transfer and release*				
Inter-prison transfer	14 (40)	16 (44)	8 (44)	38 (43)
Released during follow-up	17 (49)	6 (17)	4 (22)	27 (30)
Evidence of CMHT referral (of those released, *n* = 27)	6 (35)	5 (83)	2 (50)	13 (48)

MHA, Mental Health Act 1983; CPA, care programme approach; MHIR, mental health in-reach; ACCT, Assessment, Care in Custody and Teamwork; HSU, high secure unit; MSU, medium secure unit.

a. This included those subject to hybrid hospital orders (section 45A of MHA, *n* = 3) and those transferred to hospital for treatment from prison, either on remand/awaiting trial (ss. 48 and 49 of MHA, *n* = 11) or serving a current custodial sentence (ss. 47 and 49 of MHA, *n* = 64).

b. Two remittals were not assessed following referral, 20 remittals were deemed ‘not appropriate’ for MHIR care.

c. Five remittals were discharged from MHIR as they were deemed ‘not appropriate’, three were discharged owing to not engaging with MHIR.

d. Three stopped taking antidepressant medication (including citalopram, trazodone and sertraline); 32 stopped taking antipsychotic medication (including amisulpride, zuclopenthixol, chlorpromazine, olanzapine, quetiapine and risperidone).

e. Based only on evidence in medical documentation.

**Table 2:** Rate ratio comparisons for likelihood of persons being place under Assessment, Care in Custody and Teamwork (ACCT) during follow-up (*n* = 89): personality disorders versus all other diagnostic categories combined

	*n* (%)					
	Total	Personality disorder (*n* = 36)	Other (*n* = 53)	χ²	*P*	Rate ratio (95% CI)
Under ACCT	44 (49)	20 (56)	21 (40)	2.19	0.139	1.40 (0.90–2.18)
≥2 ACCTs during follow-up (versus 1 ACCT only)	13 (29)	10 (50)	3 (12)	7.37	0.007	4.00 (1.27–12.58)
Self-harm/attempted suicide	27 (30)	17 (47)	10 (19)	8.16	0.004	2.50 (1.30–4.82)

### Post-remittal aftercare

#### Section 117 aftercare entitlement

It was unclear in the records whether section 117 meetings took place and were attended by MHIT practitioners. Documentation of these meetings varied in quality or was not present. Most persons remitted had the legal right to continued aftercare services under section 117 of the MHA (88%, *n* = 71). Seventeen persons remitted were subject to the CPA approach at any point during their 1-year follow-up (19%), including just 14 of those with section 117 legal entitlement (20%).

#### Access to MHIT at the remittal prison

There was evidence of a referral to the MHIT for 98% of remittals (*n* = 87), the majority of which were made following the prison reception screening process (85%, *n* = 74/87). Almost two-thirds of referrals were accepted (75%, *n* = 65/87); 20 (23%) were rejected owing to the individual not meeting the service criteria, as determined by the assessor/team; 2 referred patients were not assessed. Of those accepted, 8 were discharged from the service during follow-up owing to non-engagement (*n* = 3) or not meeting the service criteria (*n* = 5). The remaining accepted patients received input from MHIT in their remittal prison, i.e. up to 1-year follow-up date or inter-prison transfer date.

#### Treatment

The majority of patients were prescribed psychotropic medication at time of discharge from medium secure services (83%, *n* = 74), almost half of whom discontinued their psychiatric medication during follow-up in opposition to clinical opinion (*n* = 35/74, 47%). This included 54% of those with a primary diagnosis of personality disorder and 48% of those with an SMI diagnosis; of these all but three discontinued prescribed antipsychotic medication (see Table 1 for a breakdown of access to professionals and treatments other than psychiatric medications).

### Post-remittal intervention

#### Assessment, Care in Custody and Teamwork (ACCT)

There were 61 recorded ACCT documents opened, relating to 44 patients (49%). The majority of these patients (*n* = 31/44, 70%) had one ACCT document opened; however, 13 of these patients (30%) had 2 or more ACCT documents opened during the follow-up period. Patients with a primary diagnosis of personality disorder were four times more likely to have had two or more ACCT documents opened during follow-up than the other groups (50 *v*. 12%, *P* = 0.007). Patients with a primary diagnosis of personality disorder were also 2.5 times more likely to have a recorded incident of self-harm or attempted suicide during follow-up than the other groups (47 *v*. 19%, *P* = 0.004). Incidents of self-harm/attempted suicide were recorded for 27 patients (30%).

#### Healthcare wing, segregation and readmission to secure care

Over one-third of patients (*n* = 33, 37%) spent time admitted to a prison healthcare wing for psychiatric assessment/treatment, 19 patients (21%) spent time in segregation for behaviour management and 19 patients (21%) were re-referred to secure psychiatric services during the follow-up period. Nine re-referrals were not accepted; however, 10 were readmitted to medium secure services during the follow-up period.

### Inter-prison transfer and release

#### Inter-prison transfer

**Fig. 2:**
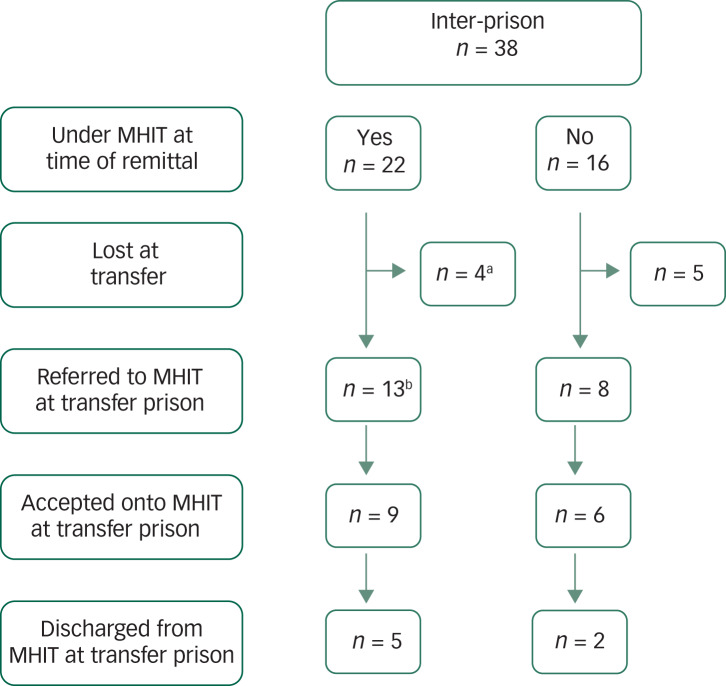
Access to aftercare following inter-prison transfer post-remittal.

Thirty-eight patients (43%) were subject to inter-prison transfer following prison remittal. The majority were transferred on one occasion during the 1-year follow-up (*n* = 30), but 8 patients were transferred more than once during this time. Just under half (45%) of prison transfers happened within the first 3 months post-remittal (Table 3). Twenty-two were under MHIT in the remittal prison but on transfer only four remained engaged with their transfer prison MHIT service at time of follow-up (Fig. 2).

MHIT, mental health in-reach team. a. 4 were referred by their remittal MHIT. b. 8 were referred by their remittal MHIT.

**Table 3:** Transfer and release additional details

	*n* (%)
Length of stay in MSU prior to remittal (*n* = 96)	
0–6 months	52 (54)
6–12 months	19 (21)
12–18 months	10 (11)
18–24 months	5 (5)
>24 months	10 (10)
Frequency of prison transfer (*n* = 89)	
One transfer	30 (34)
Two transfers	7 (8)
Three transfers	1 (1)
Time between remittal and first transfer (*n* = 38)	
0–3 months	17 (45)
3–6 months	11 (29)
6–9 months	5 (13)
9–12 months	4 (11)
Missing	1 (3)
Time between remittal and release (*n* = 27)	
0–3 months	10 (37)
3–6 months	6 (22)
6–9 months	6 (22)
9–12 months	5 (19)
Reason for remittal for prison releases (*n* = 27)	
Responsible-clinician-directed remittal (*n* = 25)	
Treated	14 (52)^a^
No SMI	2 (7)
Not engaging	6 (22)
Risk/management	3 (11)
Court-directed remittal (*n* = 2)	
Sentenced	1 (4)
Re-remanded	1 (4)
Length of stay in MSU for prison releases (*n* = 27)	
0–6 months	14 (52)
6–12 months	6 (22)
12–18 months	3 (11)
18–24 months	2 (7)
>24 months	2 (7)

MSU, medium secure unit; SMI, severe mental illness.

a. Eight were close to their earliest release date and two were working for parole.

#### Prison release into the community

Twenty-seven (30%) of those remitted were released from prison during the follow-up period, and over one-third of these releases (37%) took place within 3 months of remittal to prison (Table 3). The shortest length of time between remittal and release was 1 day. Two of those released were subject to an IPP sentence. Sixty-three per cent of prison releases were patients with a primary diagnosis of SMI, representing almost half of all remittals with an SMI diagnosis (*n* = 17, 49%). There was evidence of a referral to or liaison with a community mental health team (CMHT) for just 13 prison releases (48%). This included all but one released prisoner with a diagnosis of personality disorder, but for released prisoners with a diagnosis of SMI, just 35% (*n* = 6) were referred (Table 1).

## Discussion

This study has identified the circumstances by which NHS medium-security patients are remitted to prison. This includes court-directed remittal (17%), sometimes in opposition to clinical opinion, and remittal directed by the responsible clinician (83%). Over half of responsible-clinician-directed remittals were due to treatment completion and patients were therefore returned to continue their custodial sentence or await trial. However, 28% of patients were remitted owing to not engaging with treatment or being too ‘high risk’ to remain detained within the service. To our knowledge, this is the first documentation of this clinical practice and represents an important area for further inquiry that we plan to investigate (see ‘Implications for practice and further research’ below). An outline of future research is presented in the conclusion. Follow-up was achieved for 93% of persons remitted, and novel data on access to aftercare and early outcomes is presented. The following sections discuss these findings collectively in the context of currently policy and legislation.

### Rights to aftercare

There are both historical and environmental/cultural factors that make effective comparisons of prison mental health services with models of care available in the community problematic.^28^ However, although many aspects of care may look different from those received in the wider community, patients being cared for in the criminal justice system should have equivalence, in terms of treatment, rights and safeguards, with community patients.^29,30^ Subject to section 117 of the Mental Health Act 1983, those discharged from longer-term detention for psychiatric treatment are entitled to free aftercare services from local authorities and the NHS. Aftercare should meet the needs arising from, or relating to, mental disorder and reduce risk of deterioration, thereby reducing the risk of hospital readmission. This is facilitated through the care programme approach (CPA), with a person-centred care plan and a clinician who coordinates care. The CPA framework has been invaluable in setting principles and good practice standards as services in the community have developed.^31^ The principles of CPA have therefore been accepted as an essential foundation for improvement of the quality of community services, and patients have stressed the value of therapeutic relationships with care coordinators in enhancing recovery.^32^ The legal right to aftercare under section 117 of the MHA was relevant to 88% of the cohort, and therefore it was anticipated that the majority of people remitted would have their aftercare managed through the CPA process during follow-up. However, few patients were found to be subject to the CPA process on return to prison and just 18% of those with the legal right to aftercare had a CPA care plan in place at follow-up.

It was identified that patients were largely referred to MHITs at their remittal prisons, but around a quarter of referrals were not accepted, and some patients were subsequently discharged as they did not meet the criteria set by MHIT services. This was observed for patients across the diagnostic range, including those with a primary diagnosis of SMI. Failure to ensure adequate aftercare and such an informal discontinuation of section 117 aftercare entitlement is in breach of Article 5 of the European Convention on Human Rights and, consequently, the Human Rights Act 1998.^33^ It is therefore critical that renewed attention is given to the quality of discharge/aftercare planning for those returned to prison, to ensure that they are afforded the same rights to care and treatment as their community counterparts. Ensuring adequate and proportionate aftercare arrangements for persons remitted to prison is not only essential, but has potential to elicit wider benefits for this vulnerable group, particularly in relation to adverse events associated with loss of aftercare at this key transition point in their care, and to eventual release from custody into the community.

### Opportunities for loss of aftercare

Key transition points that resulted in loss or denial of aftercare included not only remittal to prison, but also inter-prison transfer and release from custody. Handover from the remittal prison MHIT to services based in the transfer prison was not conducted for almost half of those engaged with an MHIT at time of remittal, some of whom ceased to receive care and monitoring by mental healthcare professionals for the remaining follow-up period.

Almost one-third of persons remitted were released into the community during the follow-up period. Ten patients were documented as close to their earliest release date (ERD) or eligible for parole, at the time of their remittal. It is unclear why these patients did not remain in medium secure services until the end of their custodial sentence, which may well have ensured successful transition into the community and referral to a CMHT. Preparation for release from custody represents a challenge for MHITs and it is well-established that failure to connect with an appropriate CMHT post-release is linked to elevated mortality risk among recently released prisoners in the USA,^34^ with increased risk of relapse, violence, reoffending, suicide and other causes of death.^35^ Therefore, of particular concern are released patients who were remitted owing to engagement/risk issues. The quick release of patients who are untreated, symptomatic and therefore considered to be at elevated risk raises public protection issues. The potential for relapse or reoffending in this group is likely to be high. Collectively these findings are concerning, particularly at a time of increased media focus on what happens to patients who are released from secure psychiatric services.

### Treatment and management

We did not assess the quality of the care and treatment received by these individuals on return, but we did assess receipt of services and access to professionals. This contact was limited; around one-third were not reviewed by a psychiatrist during follow-up, and even less received care/treatment from other mental healthcare professionals (psychologists, social workers, etc.). Almost half of the patients who were prescribed psychotropic medication discontinued use in opposition to clinical opinion. For these individuals in prison, there is no process under the MHA to ensure medication/treatment adherence.

Half of the persons remitted required intervention via the Assessment, Care in Custody and Teamwork (ACCT) process, and 30% were documented as having self-harmed, particularity those with personality disorder diagnoses. This is likely an underestimation of risk across all persons remitted. Likewise, admission to prison healthcare wings for psychiatric assessment/treatment was required for over one-third of remittals. Collectively, these interventions have substantial cost implications, as enhanced observations and monitoring by both mental healthcare and prison staff are required to ensure the safety of these patients. A fifth were placed in segregation for behavioural management. Segregation is not an environment designed or appropriate for those with mental health problems, and its consequences had potential to cause further deterioration.^36–38^ It is of great concern that for many persons remitted the benefits of an admission to medium secure services may have been lost on return to prison, owing to the lack of targeted aftercare. We observed that over a fifth of persons remitted had deteriorated to the point of requiring re-referral to the secure hospital estate. These all took place in the first 9 months after remittal. Readmission to in-patient psychiatric services is often used as an indicator of quality of in-patient care and, in this case, may well represent premature discharge from medium secure services and/or lack of coordination with follow-up mental health services, particularly as over half of these patients had an initial length of stay in medium secure services of less than 6 months and just three were under the CPA process at remittal.

### Strengths and limitations

This is the first study both nationally and internationally to investigate what happens to those who are returned to prison from mental health services. Legal status and discharge circumstances are described for a population of patients returned to prison from all 33 NHS medium secure services over a 6-month period, and a 93% follow-up was achieved. Follow-up could not be conducted for those returned to a prison where the governor did not support the research or the MHIT was unable to locate the patients’ notes. Interpretation of these findings should be with the caveat that all data were extracted from medical records that may be subject to reporting error or non-reporting. When data items contradicted one another or were unavailable, collateral informants were utilised to strengthen the quality of extracted information (i.e. responsible clinicians and named nurses). All data on legal status were cross-referenced with MHA administrators.

### Implications for practice and further research

There is a current drive from the Royal College of Psychiatrists’ Quality Network for Prison Mental Health Services to standardise the CPA process within prisons, and results from its current data-gathering exercise will be available in autumn 2020.^25^ In conjunction with this, we recommend that there should be significant consideration of how the CPA process should facilitate a clear transition-of-care pathway from medium secure services to prison-based care.

Our findings highlight the importance of providing adequate discharge and transition planning and targeted follow-up services for this population, to allow the patient to complete their criminal justice pathway and to transition safely into the community on their eventual release. More robust guidance for discharge and aftercare planning procedures for persons remitted should be developed, and the subsequent responsibilities of hospital mental health services and MHITs should be better defined. This might ensure that the benefits of in-patient admission are maintained and an individual's legal entitlement to ongoing aftercare is upheld. In relation to this, we have recently secured external funding to conduct a three-phased mixed-methods investigation, with the objective of gaining a more detailed understanding of current national discharge/aftercare practices and the experiences and views of all stakeholders, including those who have previously been imprisoned. This should pave the way to the development of renewed good practice guidelines for effective remittal of prisoners requiring secondary mental health services. These findings will support the NHS duty of care to prisoners with mental disorder and will enable steps to be taken to design targeted support for this population, as already exists in the community.

## Data Availability

The authors maintain sole access to the study data. Access is ongoing while further analysis is taking place.

## Supplementary material

For supplementary material accompanying this paper visit http://dx.doi.org/10.1192/bjo.2020.62. click here to view supplementary material

## Appendix

**Appendix:** Data extracted at follow-up

Category	Details
Legal and discharge pathways	Remittal and transfer prison details Secure psychiatric hospital transfer (including referrals, assessments and transfers to in-patient psychiatric services) Prison release into the community (including evidence of liaison with community mental health services)
MHIT, treatment and access to professionals	MHIT service access (referral, assessment, acceptance and discharge) Access to healthcare professionals Treatment and services received Evidence of the CPA process during follow-up
Management interventions	Healthcare wing admissions Time spent in segregation Use of prison self-harm monitoring processes (ACCT)^a^ Documented instances of self-harm and attempted suicide^b^

MHIT, mental health in-reach team; CPA, care programme approach; ACCT, Assessment, Care in Custody and Teamwork.

a. Any prisoner identified by a healthcare or prison professional as being at elevated risk of suicide or self-harm must be managed using the ACCT procedure – a prisoner-centred, flexible care plan system.

b. As capture of this data item relied on the healthcare professional being aware of the event and recording it in the patient's record, we accept that this was likely an underestimation of these outcomes within this population. It was not possible to distinguish between self-harm and episodes with and without unequivocal evidence of suicidal intent. Therefore, these two sources of information were coalesced to a single ‘self-harm/attempted suicide’ variable.
