# Supplementary material for: Service transitions, interventions and care pathways following remittal to prison from medium secure psychiatric services in England and Wales: national cohort study
Source: BJPsych Open. 2020 Aug 3;6(5):e80. doi: 10.1192/bjo.2020.62 (PMC7453795; doi:10.1192/bjo.2020.62)
Supplement: Supplementary file 1 [file bjosup.zip › S2056472420000629sup001.docx]

| Table A.1 Mental Health Act 1983, Part III:  Sections for patients concerned in criminal proceedings or under sentence | |
| --- | --- |
| Section | Description |
| Remand to Hospital | |
| s. 35 | Remand to hospital for report on accused person’s mental condition |
| s. 36 | Remand of accused person to hospital for treatment |
| Hospital and Guardianship Orders | |
| s. 37 | Powers of the courts to order hospital admission or guardianship |
| s. 38 | Interim hospital order |
| Restriction Orders | |
| s. 41 | Power of higher courts to restrict discharge from hospital  (added to section 37 hospital order) |
| Hospital and Limitation Directions | |
| s. 45A | Power of higher courts to direct hospital admission (‘hybrid’ order) |
| Transfer to Hospital of Prisoners | |
| s. 47 | Removal to hospital of persons serving sentences of imprisonment |
| s. 48 | Removal to hospital of other prisoners |
| s. 49 | Restriction on discharge of prisoners removed to hospital  (added to sections 47 or 48) |
