# Supplementary material for: Service transitions, interventions and care pathways following remittal to prison from medium secure psychiatric services in England and Wales: national cohort study
Source: BJPsych Open. 2020 Aug 3;6(5):e80. doi: 10.1192/bjo.2020.62 (PMC7453795; doi:10.1192/bjo.2020.62)
Supplement: Supplementary file 1 [file bjosup.zip › S2056472420000629sup002.docx]

Section

at admission

Remittal circumstance

Court recall and section change during admission

**Court recall**

Re-remanded (n = 1)

Custodial sentence (n = 3)

**Court recall**

Re-remanded (n = 1)

**Responsible Clinician**

Treated (n = 2)

**Section 38** (*n* =1)

Interim Hospital Order for treatment

**Remand patients** (n = 29)

**Sentenced patients** (n = 67)

**Section 47/49** (*n* = 67)

Transfer from prison to hospital with restrictions

**Section 37n**  (*n* = 1)

Notional Hospital Order for treatment

**Responsible Clinician**

Treated (n = 34) a Not engaging (n = 15) b

High risk (n = 10)

No SMI (n = 7)

**Court recall**

Immigration Order (n = 1)

**Section 35** (*n* = 4)

Hospital Order for assessment

**Section 36** (*n* = 1)

Hospital Order for treatment with restrictions

**Section 38** (*n* = 7)

Interim hospital order for treatment

**Section 48/49** (*n* = 17)

Transfer from prison to hospital with restrictions

**Section 45a** (*n* = 2)

Hybrid Hospital Direction

**Section 35** (*n* = 1)

Hospital Order for assessment

**Section 45a** (*n* = 2)

Hybrid Hospital Direction

**Court recall**

Re-remanded (n = 1) c

Custodial sentence (n = 4)

**Court recall**

Re-remanded (n = 1) d

**Court recall**

Sentenced custodial (n = 1)

**Responsible Clinician**

Treated (n = 6)

Not engaging (n = 2)

No SMI (n = 2)

**Court recall**

Sentenced custodial (n = 3)

Eight returned to complete work to apply for parole, seven were documented as close to their Earliest Release Date (ERD)

One patient was documented as close to their ERD

In opposition to clinical opinion

In opposition to clinical opinion

a

b

c

d

*Fig3.*. Patient legal pathway under the Mental Health Act and remittal circumstances

**Responsible Clinician**

Treated (n = 2)
